# Developing the multi-dimensional mobility divide index (MDI) as a methodology to assess the accessibility level of public transport systems

**DOI:** 10.12688/openreseurope.15153.1

**Published:** 2022-12-21

**Authors:** Cino Repetto, Leonardo Benzi, Martina Bagnasco, Tally Hatzakis, Florian Brinkmann, Laura Alčiauskaitė, Alexey (Aliaksei) Andrushevich, Alexandra Koenig

**Affiliations:** 1 T Bridge S.p.A., Genova, Italy; 2 Trilateral Research, Marine Point, Ireland; 3 German Aerospace Center (DLR), Braunschweig, Germany; 4 ENIL, Brussels, Belgium; 5 iHomeLab, University of Applied Sciences and Arts, Lucerne, Switzerland

**Keywords:** accessibility, public transport, method development, people with disabilities

## Abstract

**Background**: This paper presents the development of a multi-dimensional mobility divide index (MDI) for assessing the accessibility of public transport developed using a co-design approach, directly involving end-users in the index design process. The index measures the gap that persons with disabilities feel they need to over-come to use public transport in the same way non-disabled citizens do. The MDI covers six accessibility-related dimensions: 1) safety, 2) convenience, 3) comfort, 4) affordability, 5) travel time, and 6) autonomy.

**Methods**: The method paper describes the step-by-step approach to create the MDI as a set of indicators to be rated by people with different access needs to 1) provide evidence of the main criticalities to be addressed through the design and implementation of new inclusive mobility solutions, 2) guide the design of new inclusive mobility solutions and measure their impacts, and 3) inform the transport sector encouraging positive changes in transport by providing recommendations for policy-making, new directions for service innovation, improvements and practical advice or highlighting investment priorities to pave the way for a more inclusive mobility.

**Results**: We present our findings in ways that can inform universal design and provide actionable information to researchers, policymakers, transport and urban planners, operators, and stakeholders’ representatives to promote inclusive and equitable mobility solutions for all.

**Conclusions**: Finally, we suggest follow up research and innovation, as well as recommendations for its uptake and utilisation in the pursuit of European accessibility standards and requirements for products and services in the mobility sector.

## Plain language summary

The article presents an innovative method for the assessment of the level of the accessibility of the public transport services, from the disabled users perspective.

The method has been developed from the concept of the ‘mobility divide’, that is the gap that person with reduced mobility have to overcome in order to have equal mobility opportunities. The adoption of these accessibility metrics is recommended for all kinds of urban transport services. The accuracy and transferability of the mobility divide index (MDI) has been assessed in the framework of the European Project ’TRIPS’ (TRansport Innovation for Persons with disabilities needs Satisfaction).

## Introduction

### The challenge of measuring accessibility

Put simply, “Accessibility is about getting there...easily” ( Austrian Mobility Research, 2013; ISEMOA Project: Improving seamless energy-efficient mobility chains for all. Accessibility – Why we need it), where accessibility is defined as the ease of access or how easily people can reach desired activities. It is a factor on three levels: 1) macro-level (e.g., geographical accessibility and land-use patterns in terms of location, distance etc.), 2) meso-level (e.g., availability of sustainable transport modes and service level, and 3) micro-level (e.g., occurrence of various barriers (e.g., physical barriers ( Wennberg *et al.,* 2012).

The UNIACCESS Project: Design of universal accessibility systems for public transport on Design of Universal Accessibility Systems for Public Transport highlighted the needs and issues (especially for people with disabilities) for each step of the journey, such as the preparation phase Information (timetables, fares, alternative transport); ticketing services (adapted booking and information system on tickets); safe and accessible pedestrian environment on the way to the public transport station; getting on the platforms, waiting at the bus surroundings and waiting modalities; getting to the larger terminal displays, services and assistance; gaps between the vehicle and platform, aid devices facilities, pushbuttons, driver’s awareness during boarding and disembarking the vehicle; information display system, priority signalling and seating, height of ticketing machines, and non-existence of obstacles during the journey itself.

As we can see from this list, inaccessibility can be caused by any barrier of using public transport that affects people’s motivation or capacity to travel. People’s motivation or capacity to travel has knock on effects on people’s choices in other facets of life, such as going to school or work, seeing the doctor, running errands, but also meeting friends, visiting a restaurant, a gym or cinema, or simply being outdoors; hence inaccessible transport is a basis of discrimination. 

Despite increasing awareness about the importance of equal access to public transport for all citizens, including persons with disabilities, there is still lack of metrics that can measure accessibility in ways that are meaningful to all users and reflect everyone’s struggles with transport inaccessibility in their everyday life ( Miller, 2018b). 

### State of the art/ literature review

Attempts to measure accessibility by institutional actors strive for objective and quantifiable measures of accessibility, but they are mostly excluding users from the development of such metrics ( OECD/ITF, 2019). Hence, objective metrics identify whether or not ‘an idealised average user’ can access a specific service, but not how easy it is for users of various abilities to do so. As such, they bare little relevance to actual users, particularly people with disabilities who have quite different access needs from the norm. Hence, such metrics provide little actionable information as to which gaps to address to satisfy users and decrease their perceived and lived discrimination. A number of projects have already begun to address this concern.

For example, the MEDIATE Project: MEthodology for Describing the Accessibility of Transport in Europe established a set of indicators to be used for measuring the accessibility of public transport throughout Europe. The Project addressed several stakeholders, amongst whom are ‘end-user groups’. As part of their user involvement effort, they included older and disabled people to understand the faced barriers, in all stages planning, implementation, monitoring, and evaluation. Data collection in order to quantify the fulfilment of the indicators was addressed to 25 urban public transport authorities from 15 countries ( Carvalho *et al.,* 2011). An End User Platform to elicit real users’ needs and high-quality user input in the identification of indicators and the development of the self-assessment tool for measuring accessibility was the result of this project ( MEthodology for Describing the Accessibility of Transport in Europe: Final publishable summary report).

Furthermore, the PTaccess project ( PTaccess Project: Public Transport Systems’ Accessibility for Disabled People in Europe) also attempted to measure the actual state of accessibility of urban and rural public transport systems. They analysed the accessibility of passenger information, the accessibility issues in ticketing, the accessibility of stops and stations, the accessibility of vehicles, as well as the safety and reliability of services ^10^. Research was driven by experts, rather than users. Three national experts within each country were chosen for their public transport accessibility, disability and policy knowledge and experience. Interviews were carried out at the national level in 25 EU Member States with:

- an individual of a national organisation representing disabled people;
- a representative of a public transport operator; and
- a representative of the national government.

The information derived from Interviews was complemented by intensive internet research and resulted in a good practice report covering more than 70 examples of mobility systems in various countries. Examples covered individual transport modes without taking into account multimodal aspects or the entire travel chain. Good practice has accounted for different types of disabilities (motor, visual, hearing and cognitive impairments), yet it did not involve users in their evaluation or selection.

**Figure 1.**
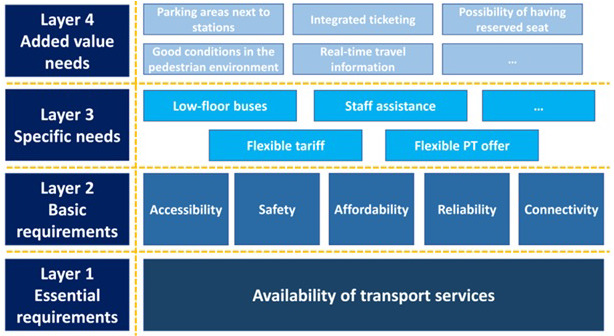
Classification of major user needs (INCLUSION Project, public, D1.3, page 43).

The INCLUSION project (2017 – 2020, https://cordis.europa.eu/project/id/770115/) focused on understanding, assessing, and evaluating the accessibility and inclusiveness of transport solutions, identifying gaps and unmet needs to ensure accessible, inclusive, and equitable conditions for all, especially vulnerable user categories. The project team analysed 51 case studies from different locations with regards to accessibility and inclusion. A methodology consisting of four layers of requirements ( Figure 1) was developed for validating the mobility needs of ‘vulnerable to exclusion’ users and population groups, based on a workshop with stakeholders and on interviews which included user groups, operators/service providers, consultancy groups, academic researchers, networking organisations and other experts.

The project gave clear recommendation to include users in the requirement specification and co-design process of mobility measures. Using such practices, a number of innovative solutions were tested and validated through real-life experiments in Innovation Pilot Labs targeting a mix of urban, peri-/sub-urban and rural areas in Belgium, Germany, Hungary, Italy, Spain, and the UK. A total of 15 innovative measures have been developed and implemented from the identified innovations addressing different transport environments, socio-economic contexts, and cultural and geographical conditions. The project proposed solutions to improve the accessibility of public transport. The solutions and measures used were based on the requirements of local users and collected via questionnaires and social media.

Table 1 contains the comparison of previously described accessibility projects regarding target group, user involvement in method design and development, as well as user involvement in method validation. Furthermore, several scientific works also address accessibility related aspects within public transportation sector developments.

**Table 1.** Comparison of different accessibility projects regarding target group, user involvement in method design and development and user involvement in method validation.

Reference project	Target group	User involvement in method design and development	User involvement in method validation
UNIACCESS Project: Design of universal accessibility systems for public transport	Policy makers /Decision makers / Government, Public administrators / Authorities, Organisations of disabled people	Initial steps during design stage	No information
MEDIATE Project: MEthodology for Describing the Accessibility of Transport in Europe	local authorities and operators (to investigate the accessibility of the public transport system)	by an End User Platform involving relevant end user organisations, including disabled people, older people and other people facing barriers to accessing urban transport	No information.
PTaccess Project: Public Transport Systems’ Accessibility for Disabled People in Europe	Policy makers /Decision makers / Government, Public administrators / Authorities, Organisations of disabled people	By including experts in interviews	No Information
INCLUSION. Project: Towards more accessIble and iNCLUSIve mObility solutions for EuropeaN prioritised areas	regulators, policy makers, stakeholder associations etc. at various levels	Users were involved in user needs classification and (partly) in the design planning of pilot tests	No information

For example, in line with their professional interests, transport planners tend to focus on the accessibility of transport modes and services (such as metros), while urban planners place more emphasis on land use metrics (such as distance to the next public facility).

According to our users, however “accessibility is a door-to-door issue”. Hence current approaches ignore the importance of multimodality. In addition, objective measures of accessibility may capture distance and travel time from a place A to a place B, ignoring other dimensions equally or even more important to users as well as individual differences between them ^12^. Other methodologies mostly identify whether or not users can access a specific service, but not how easy it is for them to do so.

There is an increasing recognition that the measurement of accessibility should consider users’ travel disutility or impedance, individuals’ travel preferences and constraints, should we want metrics to inform transport planning, operations, and policy. In addition, differences in personal needs, abilities, and travel patterns are often responsible for disparities in accessibility which can lead to unreliable analysis of transport equity ( Dixit & Sivakumar, 2020). Shedding light in such differences can yield actionable inputs for policy makers and decision makers ( Miller, 2018b). Miller (2020) summarises the issues with measuring accessibility to date. Although in his paper Miller refers to accessibility in terms of the reachability of local destinations, findings could extend to the accessibility of transportation modes (e.g., stations). The author suggests that objective models of accessibility are of little value to users’ and do not reflect their conception of utility, as well as little operational relevance in terms of the use of accessibility measures in planning analysis and decision making with regards to its economic evaluation. To increase their operational usefulness, the author suggests alternative ways of selecting of explanatory variables, levels of (dis)aggregation, treatment of travel modes, parameter estimation/specification, and travel choices and destinations, as well as a yardstick of comparison that can define meaningful thresholds for improvements in utility for users.

The literature review by Miller (2020) identified a lack of compelling, robust theoretical foundation for measuring accessibility. This lack is based on several challenges, among them: 1) there is no objective, normative standard for what constitutes ‘good’ or ‘acceptable’ accessibility, 2) the subjective (person-based) nature of most accessibility measures makes comparison of accessibility levels across groups difficult, 3) accessibility measures are ad hoc in derivation to various degrees, which makes transfer hard, 4) all conventional accessibility measures are static in nature, based on a snapshot in time, 5) the relationship between accessibility and land value is not as well established, and 6) the complexity of the concept and its measures makes accessibility very difficult for the public, politicians and even many planners to understand and use.

In a nutshell, previous projects, publications and approaches have focused on different individual aspects of accessibility, for example, travel planning and execution, including assistance and companions for disabled people, as well as the topic as a whole. In their research, policies and evaluation indicators for assessing accessibility for people with disabilities were also considered from various viewpoints.

### Research needs

Our critical evaluation of prior work highlights the lack of comprehensive, user-centric accessibility metrics that captures the main criticalities that affect users’ daily travel experience and reflects their prioritization, but are also meaningful, and easy-to-understand. The majority of previous projects did not sufficiently involve people with disabilities neither in method design and development nor method validation to ensure that the new indices represent their specific needs. In our experience, this is a critical point for meaningfully reflecting users’ perspective. It was yet clear that there is a real operational and managerial need for data and information that can inform transport investments and priorities to meet EU’s strategic priorities on accessibility as reflected on the European Commission's Study on ‘Urban Mobility – Assessing and improving the accessibility of urban areas’ ( https://transport.ec.europa.eu/facts-fundings/calls-tenders/assessing-and-improving-accessibility-urban-areas_en).

Hence, the TRIPS consortium set out to devise a brand-new measurement system for users to audit transport systems that provides a common understanding and definition of accessibility and developing a set of comparable indicators from users’ viewpoint. To this end, our effort was directed by the following three key challenges:

1. MULTIDIMENSIONALITY. The concept of ‘accessibility’ is complex and multi-dimensional. People with disabilities face simultaneously a number of interrelated factors that hinder them from travelling or at least significantly worsen their travel experience. Hence, it was necessary to establish all he factors affecting their travel and the relationships between them.
2. QUANTIFICATION. Factors affecting accessibility may stem from functional features of the transport infrastructure and/or service and can be objectively measured (e.g., distance to the nearest bus stop), it is usually users’ subjective experience of the use of the transport system that matters to them. The challenge of a comprehensive metric is to translate such personal experiences in manageable quantitative data that can drive priorities and operational decisions.
3. USER-RELATED RELEVANCE: The impact of the various transport infrastructure and/or service on accessibility may differ greatly depending on their disability. For example, the nearest bus stop may be easily accessible to the blind but really hard to get to by people on wheelchairs. Hence, accessibility depends (at least in part) from the user’s individual needs, attitudes and abilities. Moreover, it would be non-sensical and unhelpful operationally to average out their evaluations.

The goal is a new measurement system that reflects a definition of accessibility compatible with these three challenges to develop a synthetic metric that can still capture the complexity of the concept in a manageable way.

## Method

### The mobility divide index – definition and purpose

**Figure 2.**
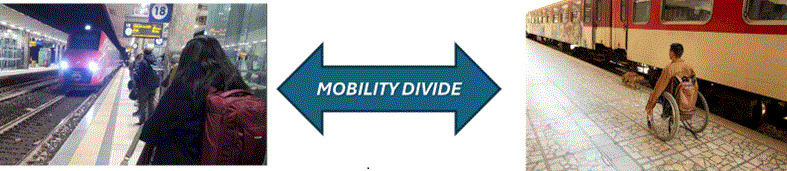
The mobility divide.

To address the goal set above, the research team developed the concept of a mobility divide, defined as “the gap that a vulnerable user has to overcome in order to have pair access opportunities to transport services, in comparison with normally endowed users” ( Figure 2). The mobility divide focuses on the user experience and on the barriers that a person with disability has to face, as a consequence of his/her conditions. Under such perspective, the level of accessibility of a transport system can be represented by the measure of the gap: the wider is the gap, the more unequal are the access opportunities for a person with disability.

The mobility divide index (MDI) has been envisioned as the composite of a set of indicators that aims to provide an overall measure of accessibility, through the evaluation of the mobility divide. The MDI multilevel structure enables a synthetic overall rating of the audited service, but also it provides different views, since it can be sorted with different keys, such as the kind of impairment or the audited transport service, so several perspectives can be made available for analysis and for a better understanding of the plus and minus of the local mobility system.

**Figure 3.**
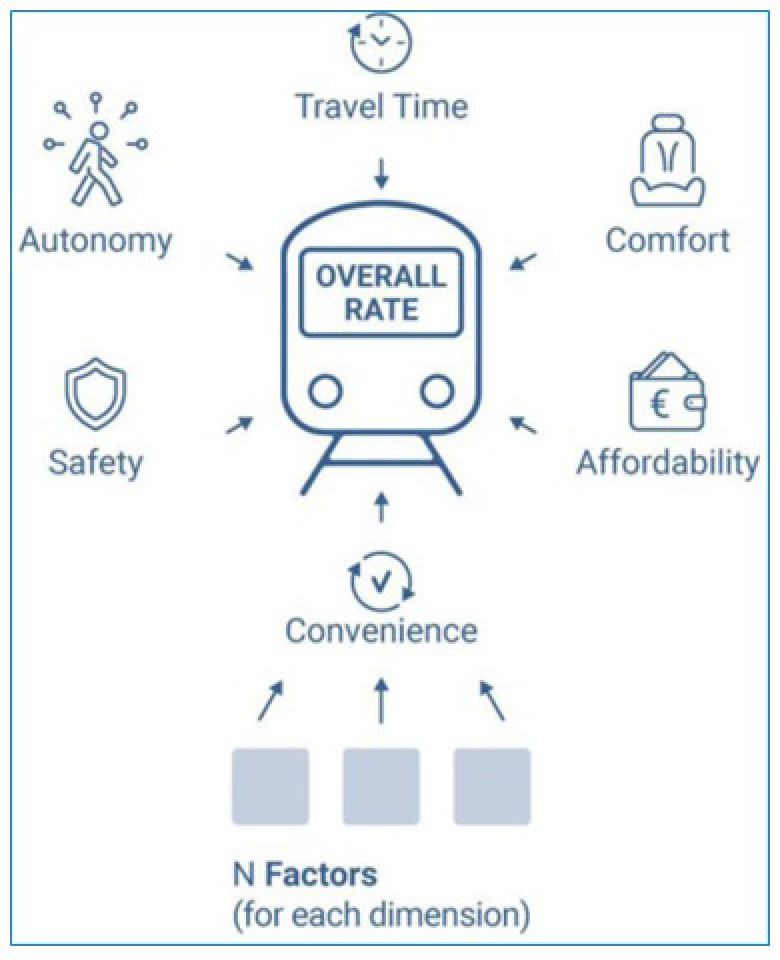
The mobility divide index (MDI) framework.

**Figure 4.**
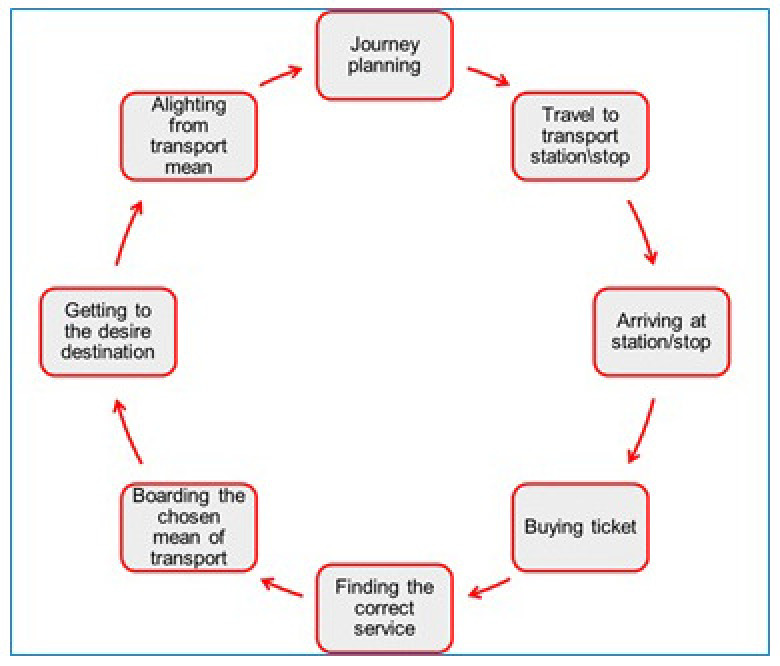
The Journey Cycle ( Lafratta, 2008).

To this end, the index is designed to be:

- Holistic, offering an easy to interpret and valid instrument to support the decision-making without dropping the underlying information base. Hence, the structure of the index has been shaped as a multi-level framework (shown in Figure 3) comprising a set of factors organised under discreet dimensions that in combination reflect different facets of the mobility divide.

- User-Centric, directly reflecting the travel experience of disabled users, and it is proposed as a mean to allow them to share their perspective on the mobility services. Through such rating scheme, they can prioritize all aspects relevant to ensure a really inclusive transport system.
- Personalisable, allowing any users to represent their subjective travel experience according to their specific impairment, expectations and attitudes and perceptions of the mobility divide.
- From users for users, involving disabled users in naming, defining of what affected their journeys (factors) and clustering and re-clustering of such items in groups according to their impact (dimensions) through an iterative, co-design process of reflecting. Such participatory approach ensures that the resulting indicators system measures accessibility in ways meaningful to users, yet useful to transport professionals.
- End-to-end, addressing all the phases of the journey experience from planning to arriving in one’s destination as per the ‘journey cycle' defined by Lafratta ( Figure 4 - Assessment of Accessibility Standards for Disabled People in Land Based Public Transport Vehicles, Lafratta (2008)).

The journey process model represents the typical phases of ‘normal’ travel; in order to deal with more complex use cases, it was integrated with further components, that can describe more optional phases, such as ‘Modal Interchange’ and ‘Disruption to Business-As-Usual’.

This allowed us to drill into the factors affecting accessibility in each phase yet being able to synthesise an index that measures the accessibility of the end-to-end journey, accounting for multimodal evaluations. The disabled users can use the MDI to audit the transport ecosystem from their own perspective, and to provide evidence on accessibility gaps and specific malfunction. Moreover, the auditing process collects data on punctual criticalities. As a result, actionable information can be provided to relevant stakeholders: researchers, transport planners, and policy makers to translate it into change actions.

Our aspiration is to establish a new common evaluation standard available for researchers, policymakers, transport and urban planners, operators, and stakeholders’ representatives to provide suggestions and recommendations to achieve more inclusive solutions in the transport sector. While the index provides user feedback on the transport system in their city, one could envision that a wide adoption of the MDI across European cities could help us take stock of the level of accessibility of the mobility systems at both the national and European level.

### Development of the MDI – the approach

**Figure 5.**
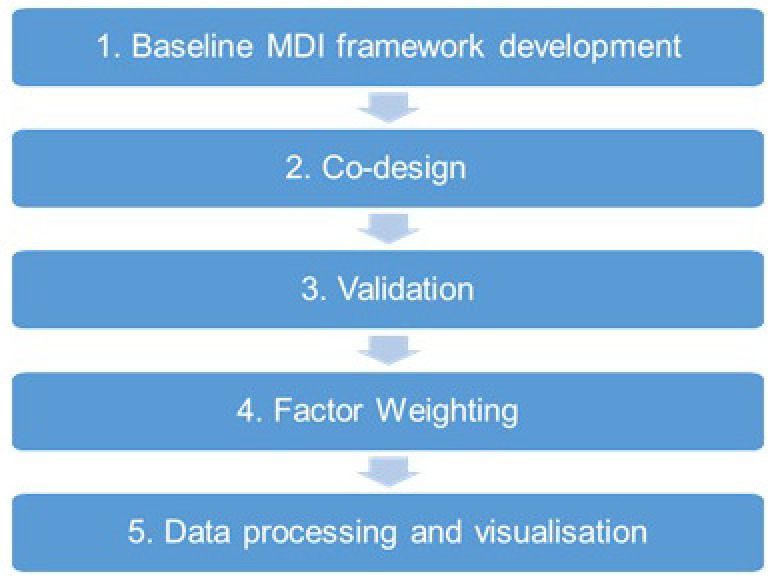
The method followed for the design of the mobility divide index (MDI).

The development of the MDI was based on a multi-step approach, visualized in the flowchart below ( Figure 5). In the following, each step of the index development is described in detail.

### Baseline MDI framework development

The index design process started by reviewing the literature on the main urban transport features that hinder people with disabilities to use public transport in the same way non-disabled citizens can. The literature analysis started in July 2020 and ended in September 2020 parallell to the writing of the TRIPS Deliverable 2.2, a report that synthesises insights gained from researcher observations and in-depth interviews with disabled people about their journeys and mobility needs, challenges and attitudes toward mobility solutions and criteria for travel, as well as their approaches to transport service barriers they have to face day by day. 

This groundwork started from the analysis of different web sources, from specialized websites, user communities, to scientific papers, European standards and best practices, and European projects. Sources were identified basing our research on the assumption that people with disabilities can face obstacles related to the following main categories: 1) accessibility to services and facilities, 2) transport organization and operation, 3) travel conditions and interactions with staff and passengers ( Bromley *et al.,* 2007; Bezzina & Spiteri, 2005; Burdett & Pomeroy, 2011; DFID High Volume Transport Policy Brief, 2019; European Commission, 2019; Geurs, 2018; Henry, 2009; Jenkins *et al.,* 2015; Lättman *et al.,* 2018; Lubitow *et al.,* 2017; Nardo *et al.,* 2018; National Aging and Disability Transportation Center: Falls Prevention Awareness in Public Transportation; Nidirect: Travelling by train; Park, 2020; Stjernborg, 2019; Sze & Christensen, 2017; Yang *et al.,* 2020).

Therefore, data were collected into different spreadsheets trying to allocate the same accessibility barriers together in the logic of developing an index. 

Data collection was enriched with findings from the Qualitative Insight Report ( Alčiauskaitė *et al.*, 2020), helped us gain local insights about accessibility in the seven project cities, and an understanding of the potential topics under consideration. These were based on a social media content analysis as well as interviews with 49 persons with disabilities. Barriers related to 1) public awareness and assistance; 2) information provision and communication; 3) infrastructure; 4) regulations; 5) vehicles; 6) stops and stations; 7) emotional barriers; 8) general service quality; and 9) COVID-19.

Content analysis of findings from both the multi-disciplinary literature research and qualitative insights report were broadly clustered according to key issue expressed around the following key dimensions that formed the baseline MDI framework for discussion with users.

- Comfort: People often experience barriers in accessing vehicles, services, and infrastructures, like toilets or ramps;
- Safety: Often, basic needs for safety are not fulfilled in public transport systems, e.g., due to the driving behaviour or a lack of safety measures;
- Convenience: Services often fail to meet the requirements of users, e.g., often high lead times for booking specialized transport;
- Travel Time: Users face challenges in using public transport due to a lack of services, e.g., missing assistance services at ramps;
- Autonomy: Often assistance services, like ordering a ramp at platforms, are essential for using public transport services, which impede independent travel.

### Co-design

This initial framework was used to engage users in reflexive discussions. It was put under scrutiny and critical evaluation in dedicated workshops. During the workshops we actively involved two representatives from ENIL and nine team leaders of the project’s expert user groups. All participants are experienced disability rights activists either persons with disabilities themselves or persons working directly with persons with disabilities. Research was conducted in line with the ethical policies of EU as described in TRIPS Grant Agreement and detailed in TRIPS DMP (Deliverables D1.5 and D1.6) and as per Deliverable 9.1. H-POPD – Requirement No. 2 describing the way TRIPS aligns with the European Commission (EC) regulations on personal data protection, all of which have been approved during the project interim review. Written informed consent was obtained from participants prior to inclusion in the study.

During the first brainstorming workshop, held on 30 September 2020, we asked participants to recount everyday barriers they face during a typical journey on the public transport services and to correlate the insights with the proposed dimensions (comfort, safety, convenience, travel time, and autonomy). This work helped attendees to better understand and give personal definitions of such concepts.

Out of the five dimensions participants agreed that travel time and autonomy can be more readily and objectively assessed through a discrete scale of levels, whereas the comfort, safety, and convenience dimensions where more open to subjective interpretation of personal experience and merited a deeper discussion. Consequently, participants were randomly assigned to different focus groups, each devoted to each of these ‘subjective dimension’.

People were asked to reflect about their dimension (e.g., comfort) in relation to each of the phases in the journey cycle ( Lafratta, 2008) (see Figure 5). All factors from all workshops in all dimensions were collected in an integrated spreadsheet (see Table 2 below) ( Benzi, 2022).

**Table 2.** Extract of the spreadsheet used in the workshop held on 30 September 2020 ( Benzi, 2022).

Evaluation criteria	Parameters	Comments or proposed changes	Additional parameters	Journey planning	Travel to transport station\ stop	Arriving at station/ stop	Buying ticket	Finding the correct service	Boarding the chosen mean of transport	Getting to the desire destination	Alighting from transport mean	Priority (from 1 to 10)
Social barriers	*feeling of* * insecurity * *caused * *by other * *passengers/* *drivers/staff * *attitudes*	*Person1.: “It is* * important to divide* * passengers/drivers* */staff attitudes”* *Person2: I'd separate * *passengers/drivers/* *staff into different * *rows here* *Person3: * *Why should one feel* *as an inconvenience?* * We have the same* *right to travel and* *need the accessibility* * which is a right? This* *should be worded as* * feeling of insecurity* * due to hinders,* *poor treatment, and* * disrespect.*	*Person2:* * In this case * *'insecurity' is* * very relate* * to comfort,* * because * *sometimes * *you feel you* * are bothering* * the staff and * *drivers*	*Person1 X* *Person2 X*	*Person1* * X* *Person * *N/A*	*Person1 X* *Person2 X*	*Person1 * *X* *Person2* * X*	*Person1 * *X* *Person2* * X*	*Person1 X* *Person2 X*	*Person1 X* *Person2 X*	*Person1 X* *Person2 X*	*Person1 * *7* *Person2 * *8* *Person3 * *10*
	*Fear of * *being an* * inconvenience* * to personnel* * and other* * passengers*											

As a take-home task, participants were asked to prioritize the comfort, safety, and convenience factors and provide comments, examples, and eventual changes about clustering and naming. A person within each participant group was selected as coordinator and tasked to integrate feedback into the master spreadsheet. Through this process, ‘affordability’ was proposed as the sixth dimension and included in the MDI.

A second validation workshop took place on 4 November 2020 with the same participants to:

1. share the outcomes of the take-home task with the other participants,
2. agree on the clustering of factors associated with each dimension and
3. approve the addition of ‘affordability’ as a key-dimension of the MDI.

### Validation

To ensure that MDI framework made sense to persons with disabilities beyond those directly involved in the development of the MDI and terms, concepts and descriptions are easily understandable in each of the languages of the pilot cities involved, we task the team leaders of the user groups to translate and engage their teams in validation of the MDI framework in their local language. Hence, the MDI was discussed offline by local user leads (LULs) with the core user teams (CUT) members, namely groups of 5–7 people with different types of disabilities who have been recruited by LULs themselves in each of the project cities. As LULs were invited to optionally present the MDI to their teams to collect their comments and opinions, results for this were available only for Brussels (French), Stockholm (Swedish), and Cagliari (Italian). Data were collected between November and December 2020 from the team leaders. Basically, people were asked to review the six dimensions and factors adding parameters and additional explanations in order to have a wider view of end-users perceptions and focus on the particularities of urban transport system that can prevent people with disabilities to not encounter difficulties during their travel experience.

People involved in this final stage of the process approved the MDI factors previously proposed by their colleagues and prioritized them. They showed that not all the MDI components have the equal importance and some interesting discrepancies exist. For example, concerning ‘Comfort’ factors, people assigned a value of 10 (on average) to getting on and off the means of transport, while using facilities and supporting infrastructure did not seem to matter the participants, as the mean value of importance rounded to 4. While such data cannot be considered statistically significant, they nevertheless highlighted the need to rate the relative importance of MDI components instead of assuming an equal rating across factors and dimensions.

**Figure 6.**
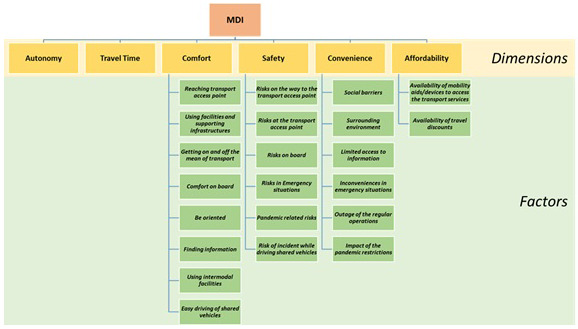
The mobility divide index (MDI) framework.

This led us to design a weighting survey describe in the *Factor Weighting* section *.* The participatory outcomes were mapped into the final MDI framework comprising a two-layer hierarchical structure of 22 factors identified by the users clustered into six dimensions (see Figure 6), defined from the users as follow:

- Autonomy: the ability to travel independently, with no need for assistance;
- Travel Time: the whole time necessary to reach destinations including extra waiting, delays, or slowdown;
- Comfort: the easy access and use of the transport services, equipment and facilities;
- Safety: the condition of not being exposed to unreasonable risks;
- Convenience: the condition of fitting in well with travellers' own needs and expectations;
- Affordability: the condition of not requiring additional expenses resulting in financial hardship.

### Factor weighting

As a composite indicator, MDI is an aggregate of all dimensions, objectives, individual indicators and variables used ( Munda, 2012). According to the OECD Handbook on Constructing Composite Indicators, weighting is a key step to building a composite indicator ( OECD: Handbook on constructing composite indicators: methodology and user guide). Weighting determines the relative contribution of particular aspects of a phenomenon over others towards the final outcome. Therefore, weights can have a significant effect on an overall indicator. No uniformly agreed methodology exists to weight individual indicators before aggregating them into a composite indicator ( COIN: Competence Centre on Composite Indicators and Scoreboards) - https://composite-indicators.jrc.ec.europa.eu/?q=10-step-guide). The existing literature offers quite a rich menu of alternative weighting methods, all having pros and cons ( Green *et al.*, 2001; Ishizaka, 2012; Moldan *et al.*, 1997; Saaty, 1977; Saaty, 1980; Saisana *et al.*, 2005b; Wind & Green, 2013). These are commonly used methods for weighting, which include the following:

- Equal weights.
- Weights based on statistical models.
- Weights based on public/expert opinion.

Equal weights would imply that all factors are of equal importance. Hence, dimensions with more variables would affect the index more which may present a biased view not representing users’ viewpoint. For this reason, the equal weighting methodology was excluded prior to our analysis. Statistical models - such as the principal components analysis (PCA) or the factor analysis (FA)- rely on large volume of quality statistical data. In such case, higher weights are assigned to statistically reliable data with broad coverage. Given that MDI is in a nascent state of development, such data does not exist. Finally, MDI aims to reflect the current users’ point of view, hence, we only examined methods based on public opinion which employ participatory methodologies in assigning weights. Researchers provide several examples of participatory methods, like the budget allocation process (BAP), the analytic hierarchy process (AHP), and the conjoint analysis (CA).

We chose the budget allocation method (NAP) as the weighting technique for estimating the dimensions weights of the MDI. Compared to the other methodologies, BAP is easier to be applied through common participatory research, as it does not require a high computational cost and process integrations with additional focus groups or workshops. For these reasons, it was selected as the most proper method to rate the relative importance of each dimension.

Based on such theory, we designed a multi-language online survey aiming to collect the importance given by European people with different access needs to the different MDI dimensions and factors. The survey was created by using Microsoft Forms and it was available in English, Italian, French, German, Greek, and Portuguese ( Benzi, 2022). An easy-to-read approach guided the design of the survey to make it understandable and accessible for all kinds of disabilities. It was launched on the 29th of January, 2021 through the ENIL newsletter and social channels. The stated goal was to reach between 100 and 150 responses.

Overall by the 10th of March, 2021, 113 persons with disabilities (as physical, sensory as visual and hearing impairment, intellectual and mental health disability) had participated in the survey expressing a rate of importance to each dimension and factor.

Results analysed through the ANOVA test (analysis of variance), indicated that prioritisation differs depending on the type of disability, phase of the journey, and mode of transport; hence reporting of data should maintain its sensitivity to reflect such differences to reflect user experiences and be useful to transport staff. This survey allowed use to derive a set of weight coefficients (w) for each factor per type of disability. A more detailed account of findings can be found at: Bagnasco *et al.*, 2021.

### Data processing and visualisation

In order to obtain each factor rating, the user (namely, every disabled person that contribute) is asked to evaluate its own level of accessibility about each factor individuated, choosing within a discrete range of fixed answers.

We have defined and agreed with the stakeholders that each answer must be expressed in natural language, where possible.

This is because firstly we would not like to require disabled users to put in an effort to assign a numerical value, and secondly, we wanted to ensure the homogeneity of the data. In this way, we have a clear and comparable scale for everyone.

In order to clarify the criteria adopted for the conversion, Table 3 below provides an example based on the autonomy dimension, for the conversion from natural language to the value of the gap. The answers are converted into a rating from –10 to 0 for each level based on the definition provided for each level, where –10 indicates the highest discrepancy with non-disabled users and 0 indicates no discrepancy with non-disabled users.

**Table 3.** Example of conversion of users’ evaluation into quantitative rating for the autonomy metric.

*Autonomy Metrics*	
USER EVALUATION	*Assigned Rating /* * Value of the Divide*
**Level 0**: the user can perform the journey step autonomously	**0**
**Level 1**: the user can perform the journey step with easy-to-use facilities (the mean of transport is provided with systems that require a minimum user effort)	**-2**
**Level 2**: the user can perform the journey step with complex facilities for disabled	**-4**
**Level 3**: the user can perform the journey step autonomously with staff assistance	**-6**
**Level 4**: the user can perform the journey step, but a caregiver is needed	**-8**
**Level 5**: the user cannot perform the journey step autonomously	**-10**

**Figure 7.**
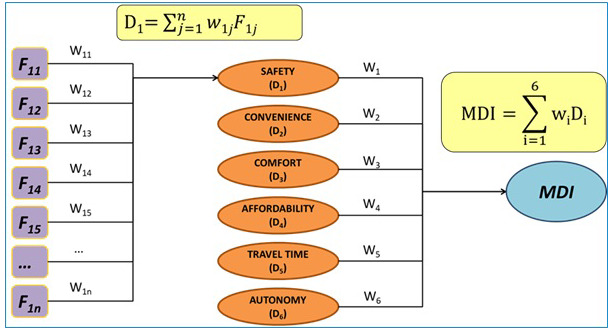
The mobility divide index (MDI) algorithm. F stands for each of the factors loading in each dimension and W stands for the weigh coefficient based on the type of disability of the person as determined in the survey expressing a rate of importance to each dimension and factor.

In order to obtain the overall index value, the MDI algorithm was calculated where each dimension value is calculated as the weighted average of its factors, and the overall MDI as the weighted average of the six dimensions (see Figure 7). This could be calculated for each individual according to his or her type of disability.

To make findings accessible and easy to understand for users and transport staff alike, a dashboard was created based on a colour coding, traffic like system.

- The green colour code represented values between 0 and -3,33 and indicates a low divide;
- the yellow colour code ranges between -3,34 and -6,66 indicates medium divide,
- the red colour code ranges between -6,67 and -10 – which indicates a high divide.

**Figure 8.**
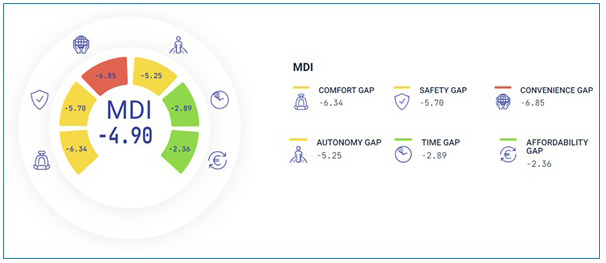
Example of visualisation of the overall mobility divide index (MDI).

**Figure 9.**
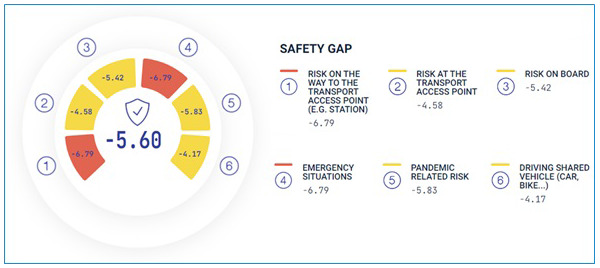
Example of visualisation of a specific accessibility dimension (Safety) and related factors.

This colour-coding system characterised both dimensions and factors and their subfactors as in Figure 8 and Figure 9 below.

**Figure 10.**
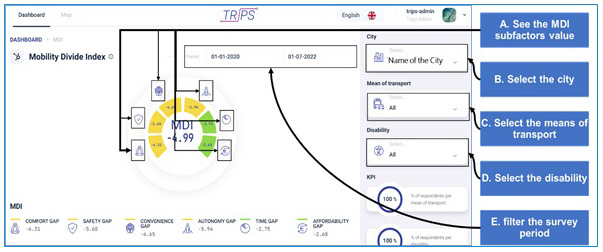
Filtering the different mobility divide index (MDI) levels.

This ‘drilling down’ organisation of the presentation of findings was chosen to facilitate users and transport staff to understand an issue but also to filter data in useful to them ways ( Figure 10).

## Intermediate results

### Expected implementation of the MDI

**Figure 11.**
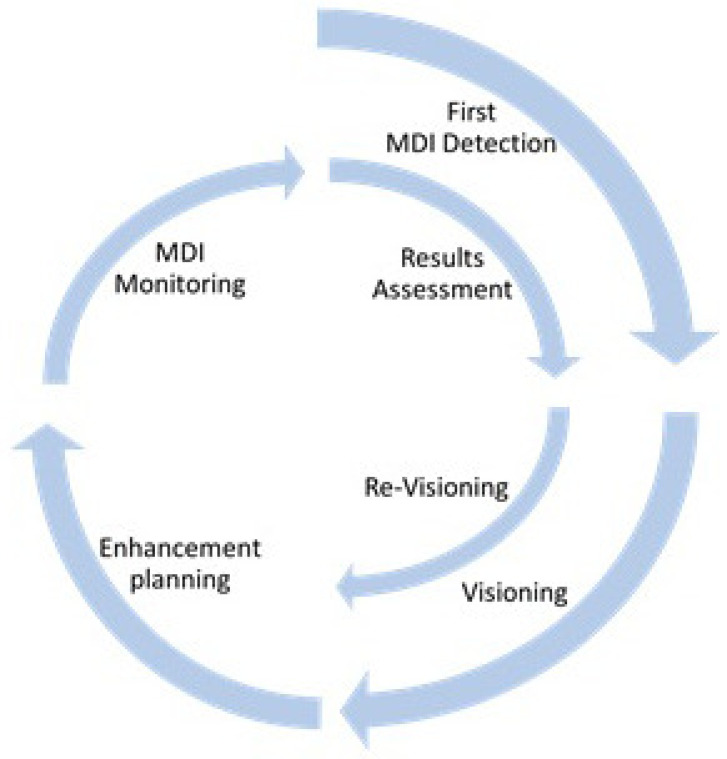
Continuous accessibility improvement through the mobility divide index (MDI) implementation.

The MDI has been conceived as a crowdsourcing method of auditing urban public transport services. In line with the notion of ‘Nothing for us, without us’, it requires the collaboration of users and institutional stakeholders in the local transport ecosystem to redress issues. MDI is expected to be used in the context of a process of continuous improvement ( Figure 11) that involves users not only in auditing but also in co-design in all the phases of the systematic redesign of a city’s accessibility, as below:

1. MDI detection: Accessibility assessment and identification of the main gaps to be tackled.
2. Visioning: Setting of priorities and of the specific enhancement objectives to be achieved.
3. Enhancement planning: definition of the improvement actions to be implemented of the reported accessibility barriers and identification of the MDI target (namely, the specific mobility gap reductions that shall be achieved).
4. MDI monitoring: continuous measurement, through the MDI, of the impact of the improvement actions, that should result in the gradual reduction of the initial gaps.
5. Results assessment: the measurement of final MDI rating should show of the satisfaction of the users’ needs and the actual improvement of the transport accessibility.
6. Re-visioning: a new improvement cycle can be launched, though the identification of further accessibility needs to be satisfied.

We envision key institutional players to be involved in the implementation of the indicator system serving particular roles, such as:

- The city authority (i.e., the Municipality, or a public mobility agency) who is in charge of the planning of mobility services and of the monitoring of the related service levels, in order to ensure the satisfaction of the citizens’ mobility needs. The MDI can represent the tool through which the city authority can check the actual accessibility of the planned transport services; therefore, they will have the role of process owner of the data collection, which will include the formal adoption of the MDI tools, the mobilization of the local disabled community in the MDI survey, and the evaluation of the audit results.
- The local transport operators, who shall commit to support the implementation of the MDI survey on the operated services. The transport operators collaborate with the city authority in the evaluation of the audit results, in the identification of the actions necessary to remove the reported barriers and in their implementation
- The community of persons with disabilities: Disability non-governmental organisations (NGOs) and private citizens are encouraged to play a leading role in the improvement process: their direct experience will be the base of the data collection for the MDI evaluation, and they will also be asked to report specific malfunctions and proposals for improvement. They shall be involved in the identification of the solutions to be implemented, in collaboration with institutional and technical stakeholders, and in the final evaluation of their effectiveness.

While the MDI proof of concept was piloted via a web survey tool, its full-scale implementation is envisioned as 1) a mobile application (TRIPS MDI – Accessible Transport Gap) that enables users to send their feedback while travelling and 2) a web dashboard (MDI DASHBOARD, available at https://mdi.tbridge.it/login) which automatically analyses and visualizes results of users’ audits. The TRIPS MDI App is available both on the AppStore ( https://apps.apple.com/lv/app/trips-mdi/id1630779990) and on Google Play ( https://play.google.com/store/apps/details?id=com.tbridge.trips), and it is used by persons with disabilities for rating the accessibility and for reporting barriers during their travel. The data collected through the TRIPS MDI App are stored in a dedicated repository and processed through the MDI algorithm. The dashboard is a public page that visualises data to make analytics useful to and actionable by policy makers, transport and urban planners, operators, stakeholders' representatives, citizens, and researchers. For example, it allows the detailed examination of specific dimensions and factors. In addition, local city authority and transport operators, have access to tools that allows them to analyse specific issues reported by the users via photos and open text.

**Figure 12.**
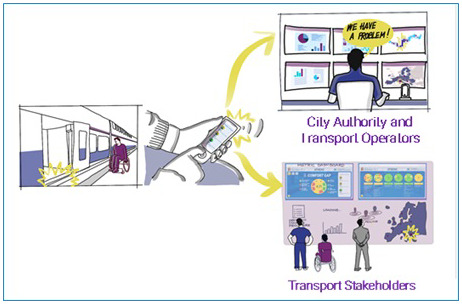
The mobility divide index (MDI) process.

 The MDI process is shown in Figure 12.

### Method validation

The MDI framework has been validated through an experimental application, that has been performed in the seven pilot cities of TRIPS Project concurrently. Representation of the local communities of disabled people were asked to use the MDI to evaluate the current accessibility level of PT in their cities, in the framework of the pilot demonstration activities of TRIPS project.

Altogether, 136 persons were engaged in the survey, and they filled an online questionnaire dealing with the different accessibility drivers. The results of the survey are reported in the following Table 4.

**Table 4.** Mobility divide index (MDI) overall value in the seven TRIPS pilot cities.

*City*	*n° Answers*	*Overall MDI value*
*Bologna*	9	-5,42
*Brussels*	8	-6,19
*Cagliari*	25	-5,88
*Lisbon*	34	-6,84
*Sofia*	8	-5,65
*Stockholm*	10	-7,28
*Zagreb*	42	-5
***Total***	***136***	

In line with the accessibility improvement cycle, the objective identification of the current level of accessibility allowed our local teams in each city to discuss the main critical points with a better understanding of the main criticalities and priorities. This helped them to better define the solutions and agree improvements and measurable targets. In our discussions the Teams’ feedback was overall positive about the process of the MDI development. In addition, no user asked for any changes in the current structure, but they provided the suggestions for the further development of the method and of the supporting tools. This set the baseline for the evaluation of implemented solution in the future, to understand the impact on improving the mobility divide.

## Discussion

The TRIPS consortium set out to develop a synthetic metric of accessibility that reflects users’ viewpoint and can capture the complexity of the concept in a manageable way. To do so, we worked with users to develop a ground up six-dimensional framework encompassing each encompassing a number of factors important to users’ and supportive of independent travel. As such we not only respected the need for multidimensionality but also the need to provide a common understanding and definition of accessibility from users’ viewpoint which in our view should be the one driving institutional actions and priorities to have real social impact. We also ensured that data can be quantified in meaningful to the users' and constructive ways to make sure information is actionable and hence user feedback can lead to constructive interactions with institutional actors and common grounds in prioritisation, investment and action. The MDI algorithm used to analyse data take into account the differences in the prioritisation of factors per type of disability to ensure that the overall MDI does not glaze over such differences but can better reflect users’ views. We have developed the MDI framework working closely with users to choose the wording and definition of factors, ensure the clustering is meaningful and reflecting of their experience and the framework covers their end-to-end travel from planning to arriving at one’s destination to reflect accessibility as an end-to-end, multimodal issue which is important to them. 

To our knowledge this is the first attempt to develop a ground-up accessibility index using a co-design approach seeking to reflect the views and sentiment of people with disabilities.

One of the key lessons learned was that close interaction with teams of persons with disabilities can facilitate a comprehensive evaluation of public transport accessibility as an end-to-end issue, by understanding the factors affecting their travel experience in each phase. Another key lesson was the necessary attention to wording and definitions during the development process to make the index relatable and relevant to users. This was important both in the English version and for its translation to user’s local languages. This is an important point for anyone who attempting to develop an index of accessibility and points to the need for user validation at its development phase. 

For the MDI to become an effective tool for policymakers, transport, and urban planners or operators and stakeholders’ representatives, it needs to be credible that it accurately reflects user opinions and provide timely and actionable information to inform policy, investments, and operational strategies. Hence, the consortium plans the wider validation of the MDI and the engagement of external stakeholder groups in its validation, testing and fine-tuning to be used by others beyond the boundary of the project. A keen interest of the stakeholders is the proof of the need of changing the approach towards the accessibility and the way to measure it. This has been confirmed by a close interest from external stakeholders (e.g., POLIS network) and disability NGOs who participated in the weighting survey.

We appreciate that this MDI effort should be put to further testing, validation, and evaluation with wider communities of users and institutional actors before it can be consolidated and established as an industry standard, but also that priorities will change as transport systems evolve and users’ abilities to navigate the transport transform due to adjacent factors, e.g., personal technologies. To enable our model to be piloted and replicated in and by other cities, both the TRIPS MDI app and the MDI dashboard can be set up for any city interested in the adoption of the MDI method. Currently, such tools are in BETA testing phase (Oct-Nov, 2022) in the TRIPS pilot cities, to finetune the applications and its adoption process. Subsequently, a new release of the applications will be launched for further piloting in European cities.

At a systemic level, the TRIPS consortium works closely with its partner UITP ( https://www.uitp.org/) and other institutional and political actors to agree on the establishment of a European Accessibility Observatory that can act as a transparent, two-way communication platform between citizens and the transport ecosystem. The platform will collect MDI data and reported barriers logged as incidents from users in each European city to monitor the level of accessibility of their transport cities at real-time and provide detailed info on the issues to be addressed. In addition, it will publish each city’s MDI dashboard, as well as institutional responses and their status by the responsible institutions. Time series data analytics will demonstrate the impact of such institutional responses on accessibility and citizen satisfaction over time. Collecting such data across European cities will provide a database of context specific information on accessibility barriers to be mined for uncovering effective best practices to address them and their principles to guide policy and practice.

## Conclusions

The paper presented the development of a multi-dimensional mobility divide index (MDI) for auditing the accessibility of public transport as the perceived disparity in the ease of using transport between disabled and non-disabled citizens. The MDI was developed using a co-design approach, directly involving end-users. We argued to the superiority of such an approach in terms of being holistic due to multidimensionality, usefulness in terms of translating user data into useful and actionable quantitative data for institutional users to enact upon yet remaining representative of user viewpoints and relevance to the users and their issues enabling meaningful representation and enabling institutional responses to be relatable from users’ standpoint. Six dimensions transpired as most relevant and important for accessibility according to users: 1) safety, 2) convenience, 3) comfort, 4) affordability, 5) travel time, and 6) autonomy and measures. We present our findings and links to tools in development in the hope that they can be replicated and utilised by institutional stakeholders to promote inclusive and equitable mobility solutions for all and the pursuit of European accessibility standards and requirements for products and services in the mobility sector. The consortium will remain open to expressions of interest by institutional actors to enable their adoption in their urban environment throughout the duration of the TRIPS project until January 2023. Into the future, we offer the mobility divide index as a part of our contribution towards a better understanding of users’ need and requirements towards a assessable and inclusive transportation for all.

## Data Availability

Zenodo: Development of the multi-dimensional Mobility Divide Index as a methodology to assess the accessibility level of public transport systems. https://doi.org/10.5281/zenodo.7300150. ( Benzi, 2022). This project contains the following underlying data: Mdi survey.xlsx (raw data of the first dissemination of the MDI survey in Task 6.1 in original language and translations). Weights.xlsx (raw data and first elaboration of MDI weights). Insecurity Level_collected comments focus group.xlsx (Insecurity comments as part of 04 Nov 2021 Workshop, screenshot seen in the Table 2) Zenodo: Development of the multi-dimensional Mobility Divide Index as a methodology to assess the accessibility level of public transport systems. https://doi.org/10.5281/zenodo.7300150. ( Benzi, 2022). This project contains the following extended data: TRIPS MDI Survey - Google Forms.pdf (widespread survey, version to be completed in English, Italian, Belgian, Swedish, Portuguese) TRIPS MDI Survey - Google Forms - bulgarian.pdf (Survey, also in Bulgarian) Data are available under the terms of the Creative Commons Attribution 4.0 International license (CC-BY 4.0).
