# Supplementary material for: Developing the multi-dimensional mobility divide index (MDI) as a methodology to assess the accessibility level of public transport systems
Source: Open Res Eur. 2022 Dec 21;2:143. [Version 1] doi: 10.12688/openreseurope.15153.1 (PMC10521049; doi:10.12688/openreseurope.15153.1)
Supplement: Peer review history

# Reviewer report: Reviewer response for version 1

**DOI:** 10.21956/openreseurope.16391.r33214

**Published:** 2023-09-14

**Authors:** Evelina Budilovich

**Affiliations:** 1 Transport and Telecommunication Institute, Riga, Latvia

The article Developing the multi-dimensional mobility divide index (MDI) as a Methodology to Assess the accessibility level of Public transport Systems provides an overview of the accessibility indicators, especially for people with reduced mobility. The state-of-the-art or literature review describes the projects and activities conducted in this field. The article is written in a sufficient scientific manner. The theme is actual, and the provided analysis method is good enough.

 The major research point is the provided methodology with the description of the method and analysis with results validation. Moreover was developed the co-design approach, which directly involves the end-users. For results validation, the mobility divided index framework was done, defined by the users and after the budget allocation method as the weighting technique was chosen for estimating the dimensions weights.

 A minor point could be formatting, such as citing the project's name (title) in the Introduction needing improvement. And to providing the reading more comfortable, the Headings numeration (2.1...) is required.

 The article is approved.

Are sufficient details provided to allow replication of the method development and its use by others?

Yes

Is the description of the method technically sound?

Yes

Are the conclusions about the method and its performance adequately supported by the findings presented in the article?

Yes

Is the rationale for developing the new method (or application) clearly explained

Yes

If any results are presented, are all the source data underlying the results available to ensure full reproducibility?

Yes

Reviewer Expertise:

Urban mobility, transport simulation, sustainable transport system development and analysis

I confirm that I have read this submission and believe that I have an appropriate level of expertise to confirm that it is of an acceptable scientific standard.

# Reviewer report: Reviewer response for version 1

**DOI:** 10.21956/openreseurope.16391.r30529

**Published:** 2023-01-05

**Authors:** Nicola Valentino Canessa

**Affiliations:** 1 Department Architecture and Design (DAD), Università di Genova, Genoa, Italy

The article is of great interest because it summarizes all the general parts of TRIPS-funded research. Of good generosity are the cross-references to previous research and references that allow one to move around within the topic in order to explore it further and creating a good steering scheme of the research presented in the article.

 Obviously this scholarly article is the narrative of the funded research, and in its main body I think there is no point in asking for any changes or insights that would be outside the research itself that has already been accomplished, because perhaps the only weakness is the number of questionnaires produced, which are already a good sample, but perhaps do not allow for an appreciation of all the facets that the MDI could provide with respect to the authors' suggestions.

 Perhaps in the main body it could be explicated with more practical examples of how disabled users can interact through the mobile application and provide the correct (and subjective) data to populate the index data and whether and how the data user can, as I imagine, act site specific.

 The data present and attached are of great interest and complete, I also consider for reproducibility by other researchers, and for this reason I believe it is not necessary to upload other data (application-related, for example) that would have necessary copyright coverage.

 Not only do I find the article in its structure very interesting, but especially the research presented and the presentation of MDI.

Are sufficient details provided to allow replication of the method development and its use by others?

Yes

Is the description of the method technically sound?

Yes

Are the conclusions about the method and its performance adequately supported by the findings presented in the article?

Yes

Is the rationale for developing the new method (or application) clearly explained

Yes

If any results are presented, are all the source data underlying the results available to ensure full reproducibility?

Yes

Reviewer Expertise:

Researcher in Urban Planning (UNIGE), my fields of research include new technologies for city management and urban transportation.

I confirm that I have read this submission and believe that I have an appropriate level of expertise to confirm that it is of an acceptable scientific standard.
